# Supplementary material for: UPF1/SMG7-dependent microRNA-mediated gene regulation
Source: Nat Commun. 2019 Sep 13;10:4181. doi: 10.1038/s41467-019-12123-7 (PMC6744440; doi:10.1038/s41467-019-12123-7)
Supplement: Supplementary file 1 — Supplementary Information [file 41467_2019_12123_MOESM1_ESM.pdf]

Supplementary Information for
UPF1/SMG7-dependent MicroRNA-mediated Gene Regulation

Park *et al.*

Supplementary Figure 1. Related to Figure 1

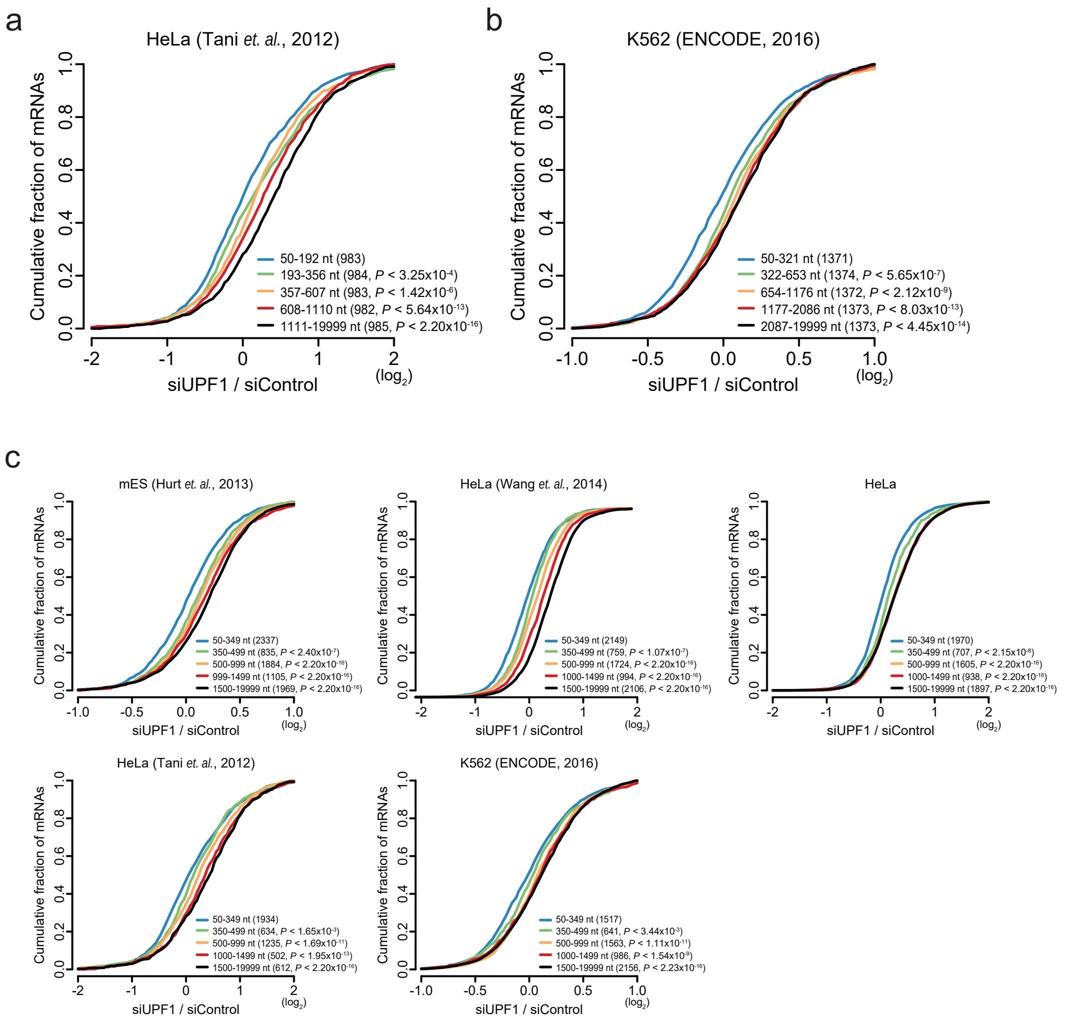

Supplementary Figure 1. (a, b) CDF graphs of Tani et al. (2012) HeLa (a) and ENCODE K562 cell data (b). (c) CDF graphs of public data and our data with the same bin sizes as in previous studies. Otherwise, as in Fig. 1b–d.

Supplementary Figure 2. Related to Figure 2

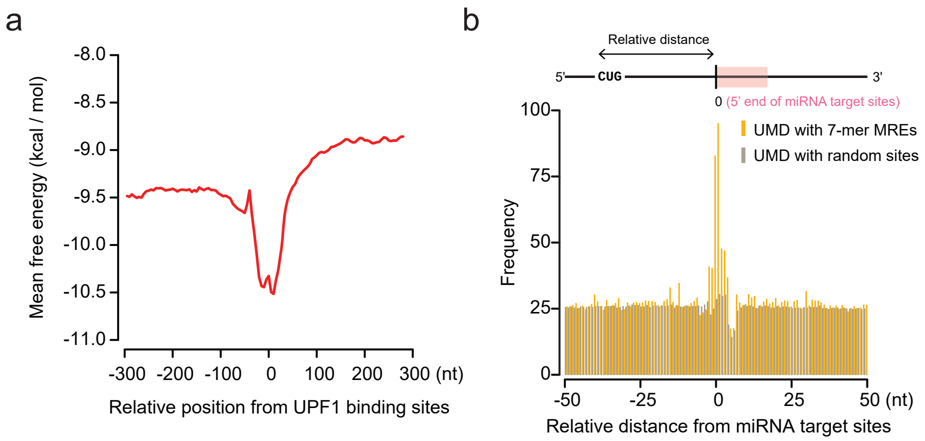

Supplementary Figure 2. (a) Landscape of mean free energy near UPF1-binding sites, determined from UPF1 CLIP-seq data (mES cells). (b) The frequency of CUG triplet nucleotides near the 7-mer sites of UMD targets are shown as bars (orange for miRNA 7-mer MREs; gray for random sites).

Supplementary Figure 3. Related to Figure 3

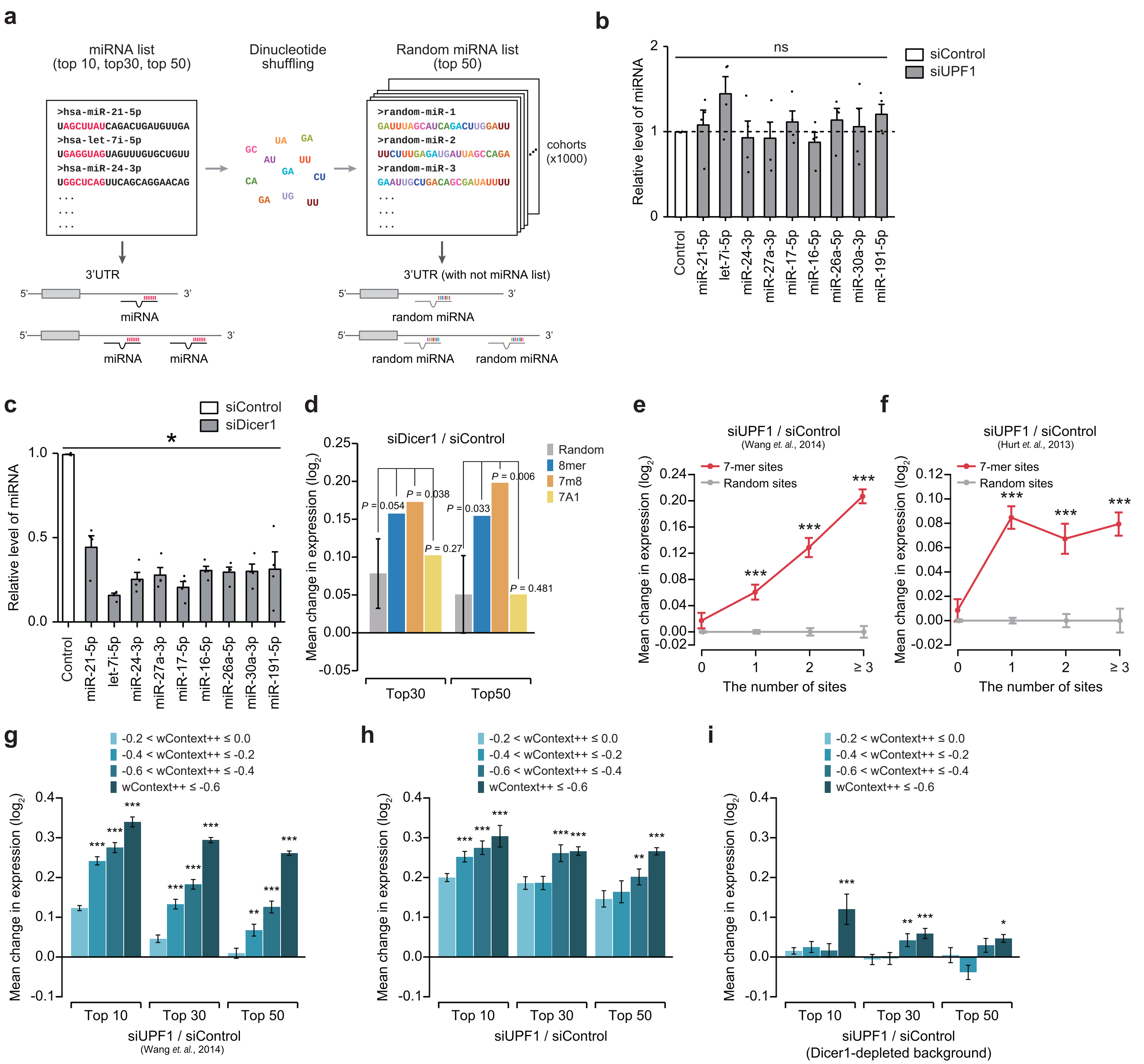

Supplementary Figure 3. (a) Schematic flow of the generation of random miRNAs and corresponding random target sites (related to “Generation of random controls and statistical tests” in the Materials and Methods section). (b, c) Relative amounts of miRNAs were quantified by RT-qPCR in the presence of siUPF1 (b) or siDicer (c). (d) Changes in the expression of miRNA targets under Dicer1 depletion are shown in bar graphs. (e, f) Mean changes in expression of dEJ-free mRNAs with different numbers of miRNA 7-mer sites are shown over Wang et al. (2014) data (e) and Hurt et al. (2013) data (f). Otherwise, as in Fig. 3f. (g-i) Mean changes in expression of dEJ-free mRNAs with 7-mer sites of the 10, 30, and 50 most abundant miRNA families in response to UPF1 depletion (Wang et al., 2014 data for (g); our data for (h)) and in response to UPF1 depletion in the Dicer1 depletion background (i) are shown over different wContext++ score bins. The levels of miRNA were normalized to that of U6 snRNA. Mean values and standard errors were calculated from independent experiments (*, *P* < 0.05; unpaired Student’s t-test; ns: not significant). The minimum number of independent biological replicate experiments was *n*=4 in (b and c).

Supplementary Figure 4. Related to Figure 4

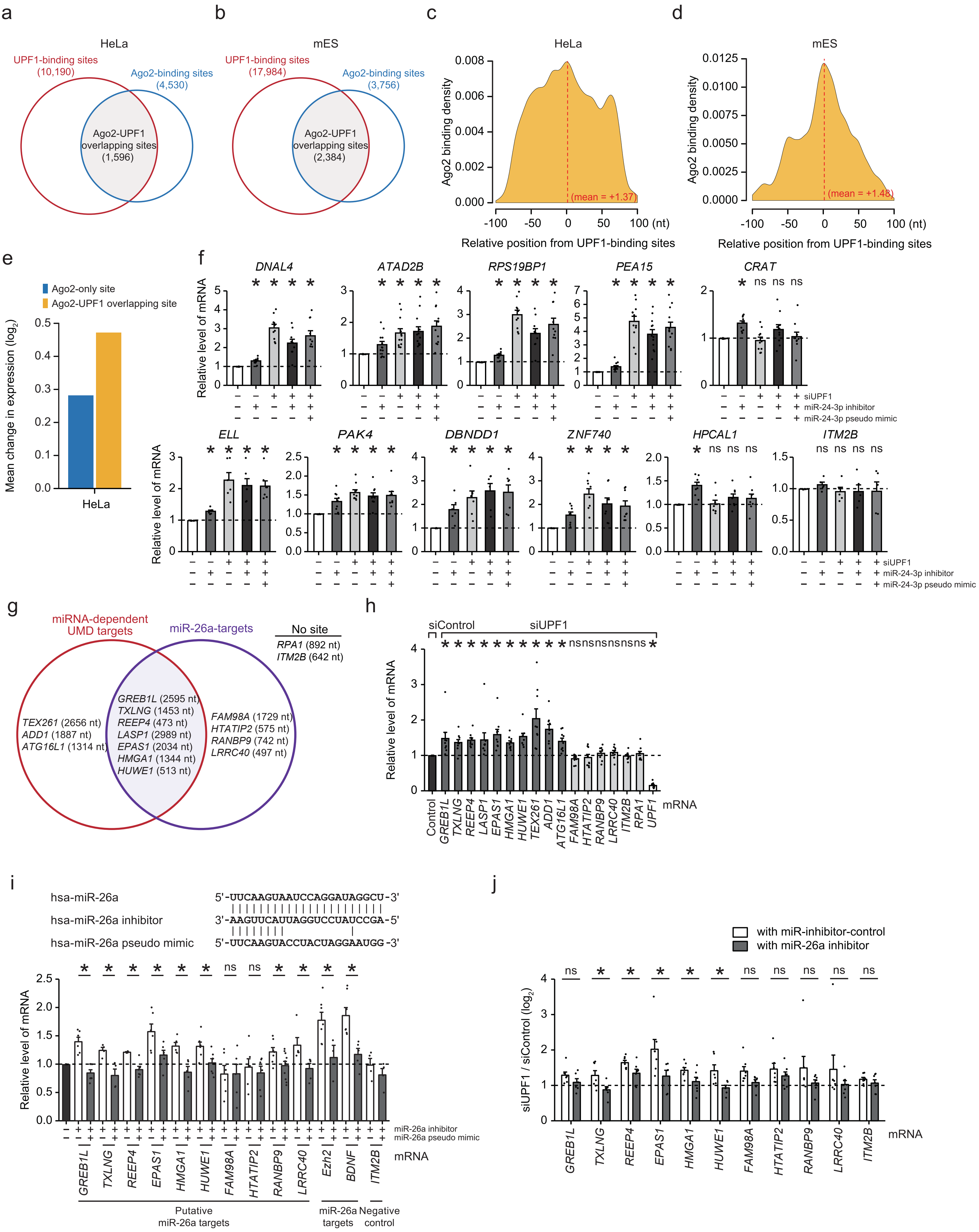

Supplementary Figure 4. Related to Figure 4

k

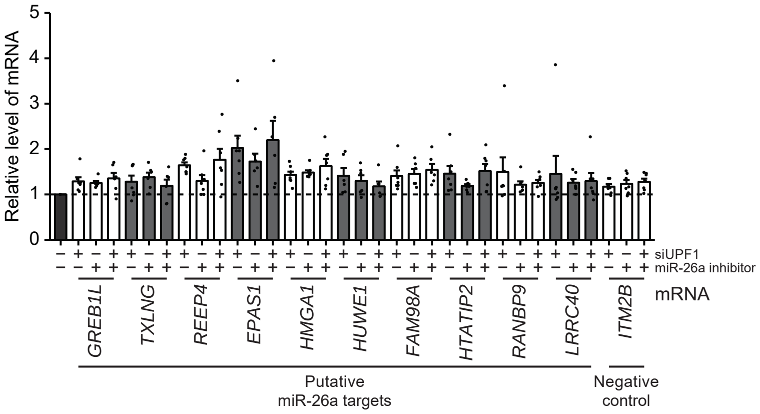

Supplementary Figure 4. (a, b) Venn diagrams of UPF1-binding sites and Ago2-binding sites from evident CLIP-seq data in HeLa (a) and mES cells (b). Numbers in parentheses are detected sites in the 3′UTR from CLIP-seq data. The shared area indicates the overlapping sites of Ago2 and UPF1. (c, d) Density plot of Ago2-binding sites near UPF1-binding sites in HeLa (c) and mES (d) cells. The dotted red line indicates the mean position of the density plot. (e) Mean changes in the expression of dEJ-free mRNAs that embed Ago2-only sites (blue) or Ago2-UPF1 overlapping sites (orange) in response to siUPF1 treatment in HeLa cells. (f) Comparisons of transcript levels in the presence or absence of siUPF1 or the miR-24-3p pseudo-mimic or miR-24-3p inhibitor, as shown in Fig. 4b and 4f, displayed by transcript. (g) miR-26a-dependent UMD and non-UMD targets are depicted in the Venn diagram. No site denotes mRNAs without sites of the 10 most abundant miRNA families. Numbers in parentheses are the 3′UTR lengths of each transcript. (h) Upregulated mRNA levels obtained from RNA-seq results in UPF1-depleted cells were confirmed by RT-qPCR. The levels of mRNA were normalized to that of GAPDH mRNA. (i) Sequences of the human miR-26a, miR-26a antisense inhibitor, and miR-26a pseudo-mimic used in this study (upper panel). HeLa cells were transfected with the inhibitor and/or pseudo-mimic, and RT-qPCR was performed to quantify the level of miR-26a targets. The levels of mRNA were normalized to that of GAPDH mRNA (bottom panel). (j) Similar to Fig. 4f; however, the levels of transcripts regulated by miR-26a were examined. The levels of mRNA were normalized to that of GAPDH mRNA. (k) Comparisons of transcript levels with or without treatment with siUPF1 or miR-26a inhibitor, as in Supplementary Fig. 4j, displayed by transcript. Mean values and standard errors were calculated from independent experiments. Asterisks denote statistically significant differences (*, $P < 0.05$; unpaired Student’s t-test); ns, not significant. The minimum number of independent biological replicate experiments was $n \geq 3$ in (f, h-k).

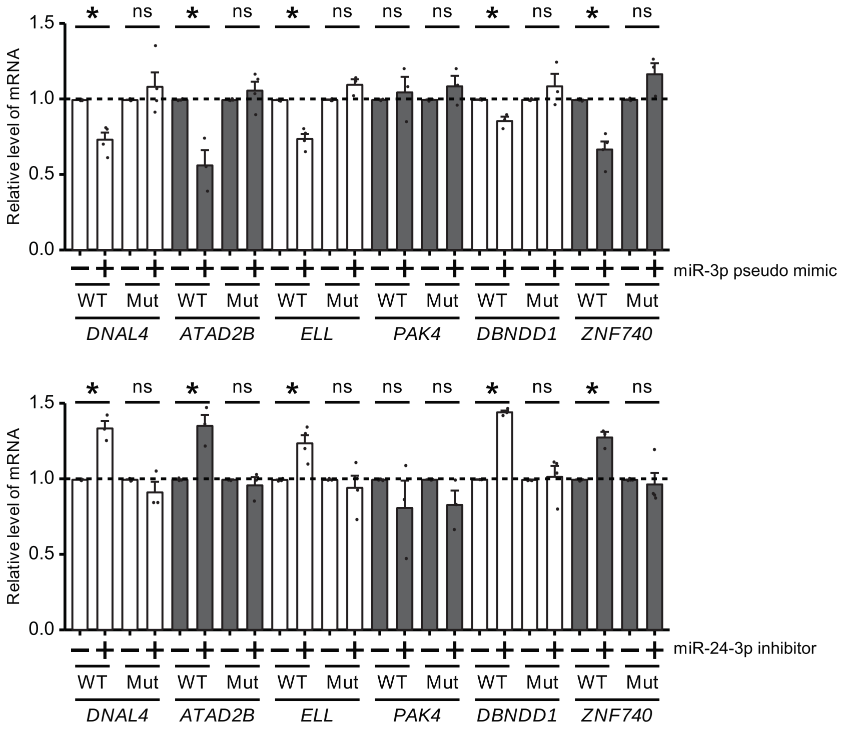

Supplementary Figure 5. The putative binding sites and mutated sites of the miR-24-3p targets were inserted, as depicted in Fig. 5e. HeLa cells were cotransfected with reporter constructs with an miR-24-3p pseudo-mimic or inhibitor. RT-qPCR was performed to quantify the relative levels of transcripts. The level of FLuc mRNA was normalized to that of RLuc mRNA. Mean values and standard errors were calculated from independent experiments. Asterisks denote statistically significant differences (*, P < 0.05; unpaired Student's t-test; ns: not significant). The minimum number of independent biological replicate experiments was $n \geq 3$.

Supplementary Figure 6. Related to Figure 6

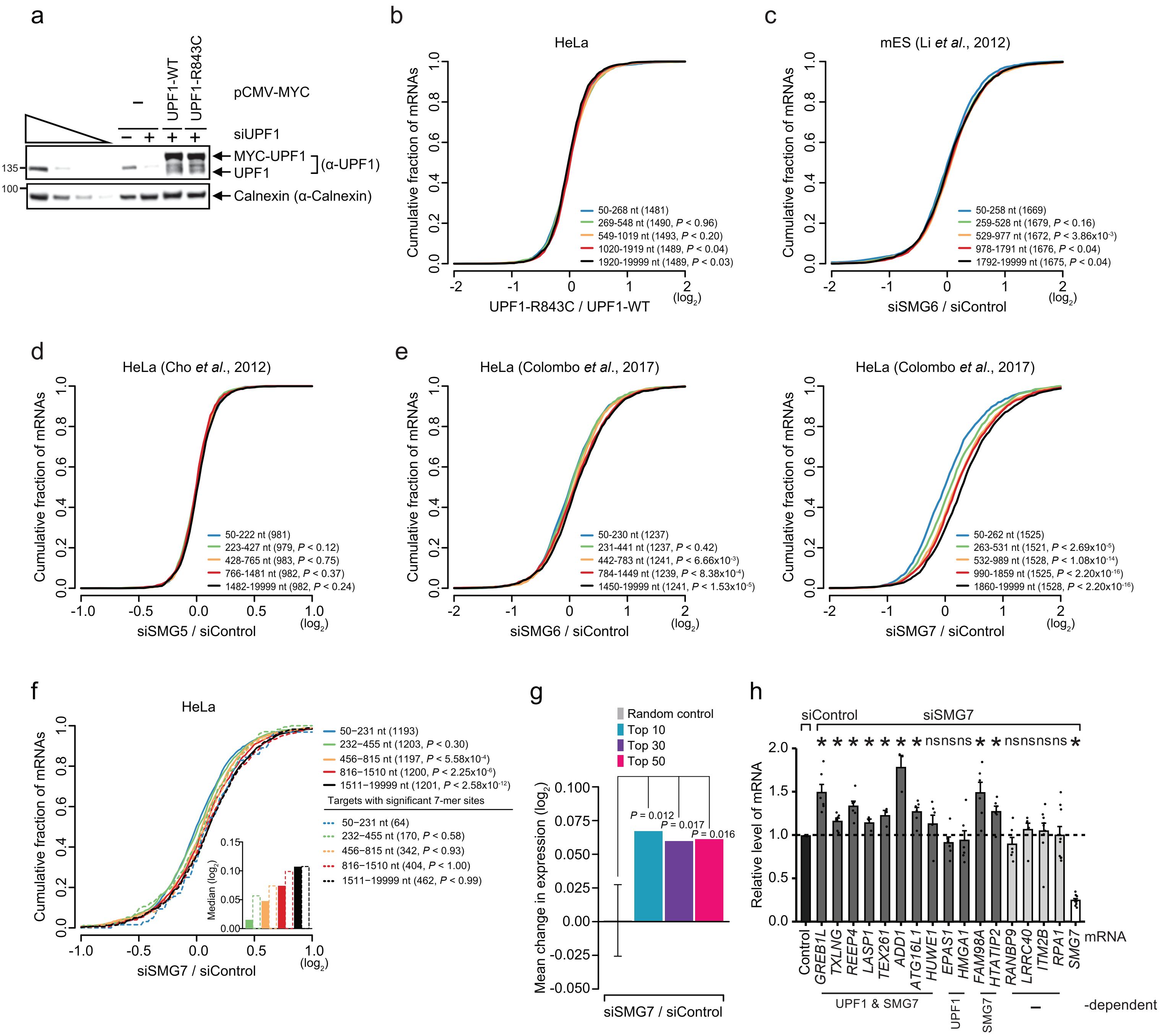

Supplementary Figure 6. (a) HeLa cells were cotransfected with siUPF1 and siUPF1-resistant MYC-UPF1-WT or R843C helicase mutants. Western blotting was performed to observe endogenous UPF1 and exogenous MYC-tagged UPF1. Calnexin served as a loading control. The three lanes show 3-fold dilutions of lysates to demonstrate that the western blotting conditions are semiquantitative. (b) CDF graphs of changes in expression of dEJ-free mRNAs in UPF1-R843C mutant versus wild-type HeLa cells are shown over different 3′UTR length bins. Otherwise, as in Fig. 1b–d. (c) CDF graphs of changes in expression of dEJ-free mRNAs in siSMG6-treated and siControl-treated mES cells are shown over different 3′UTR length bins. Otherwise, as in Fig. 1b–d. (d) CDF graphs of changes in expression of dEJ-free mRNAs in siSMG5-treated and siControl-treated HeLa cells are shown over different 3′UTR length bins. (e) CDFs of changes in expression of dEJ-free mRNAs in siSMG6-treated (left) and siSMG7-treated (right) cells versus those of siControl-treated (scrambled siRNA) cells are shown. Otherwise, as in Fig. 1b–d. (f) As in Fig. 6b, CDF graphs of changes in the expression of dEJ-free mRNAs (solid lines) and UMD targets with significant 7-mer sites (dotted lines) in RNA-seq data prepared from siSMG7-treated and siControl-treated cells. Otherwise, as in Fig. 6b. (g) Mean changes in expression (log2 scale) in the random control and UMD targets embedding 7-mer target sites of the 10 (blue), 30 (purple), and 50 (red) most abundant miRNA families under siSMG7-treated conditions. The P values were calculated by comparing the mean changes in the expression of the UMD targets with those of the random controls embedding 7-mer sites of the random miRNAs using the one sample t-test. Otherwise, as in Fig. 3a. (h) HeLa cells transfected with siSMG7 or siControl were subjected to RT-qPCR to ensure the upregulation of miR-26a targets. The levels of mRNA were normalized to that of GAPDH mRNA. Mean values and standard errors were calculated from independent experiments. Asterisks denote statistically significant differences (*, $P < 0.05$; unpaired Student's t-test); ns, not significant. The minimum number of independent biological replicate experiments in (h) was n=9.

Supplementary Figure 7. Related to Figure 7

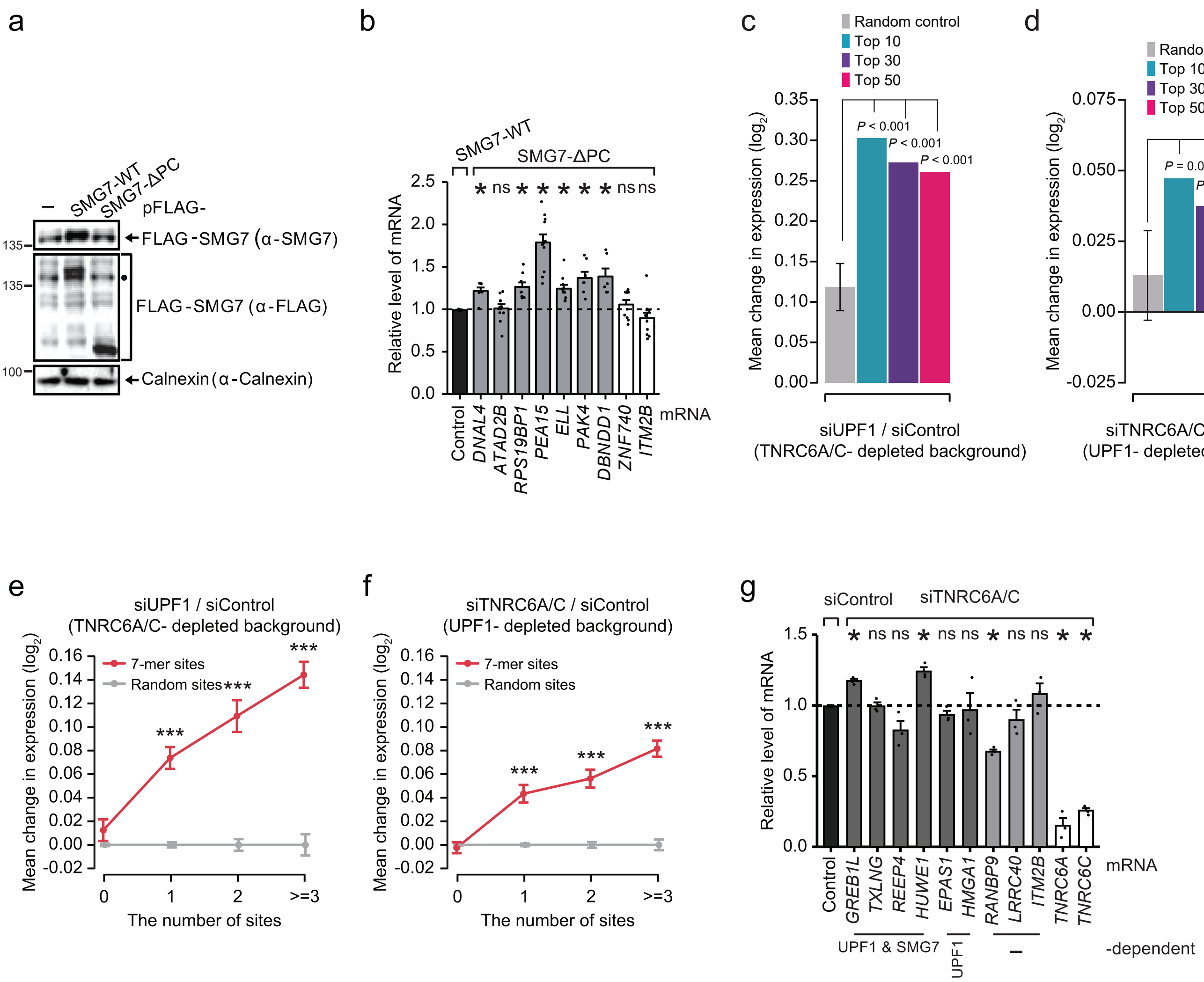

Supplementary Figure 7. (a, b) HeLa cell lysates, which were transiently transfected with pFLAG, pFLAG-SMG7 wild-type (WT) or pFLAG-SMG7 mutant (ΔPC), were used for western blotting (a). A dot represents an unspecific band. The relative transcript levels were observed by RT-qPCR (b). (c, d) Mean changes in the expression of dEJ-free mRNAs with 7-mer sites of the 10, 30, and 50 most abundant miRNA families in response to UPF1 depletion in the TNRC6-depleted background (c) or TNRC6 depletion in the UPF1-depleted background (d). P values were calculated by comparing the mean changes in the expression of those with the dEJ-free mRNAs (random controls) embedding 7-mer sites of random miRNAs using the one sample t-test. Otherwise, as in Fig. 3a. (e, f) Mean changes in expression of dEJ-free mRNA families in response to UPF1 depletion in the TNRC6-depleted background (e) or TNRC6 depletion in the UPF1-depleted background (f) are shown over different numbers of 7-mer sites of the 10 most abundant miRNA families. Otherwise, as in Fig. 3f. (g) Endogenous TNRC6A and 6C were depleted using siRNA. miR-26a target transcript levels were quantified by RT-qPCR. The mRNA levels were normalized to that of GAPDH mRNA. Mean values and standard errors were calculated from independent experiments. Asterisks denote statistically significant differences (*, $P < 0.05$; unpaired Student's t-test); ns, not significant. The minimum number of independent biological replicate experiments was $n \geq 6$ in (b) and $n=3$ in (g).

Supplementary Figure 8. Related to Figure 7

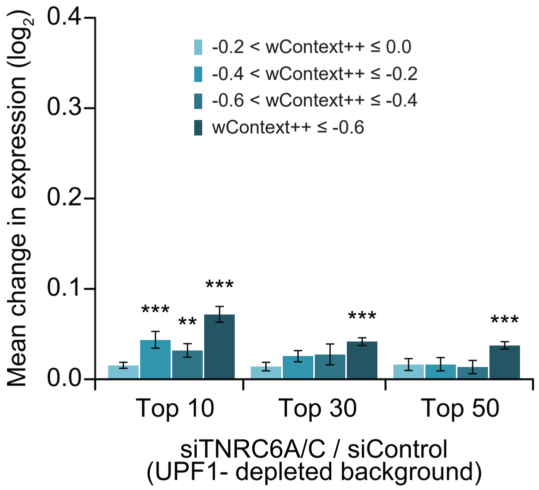

Supplementary Figure 8. Mean changes in expression of dEJ-free mRNAs with 7-mer sites of the 10, 30, and 50 most abundant miRNA families in response to TNRC6 depletion in the UPF1-depleted background are shown over different wContext++ score bins. Otherwise, as in Fig. 7h.

Supplementary Figure 9.

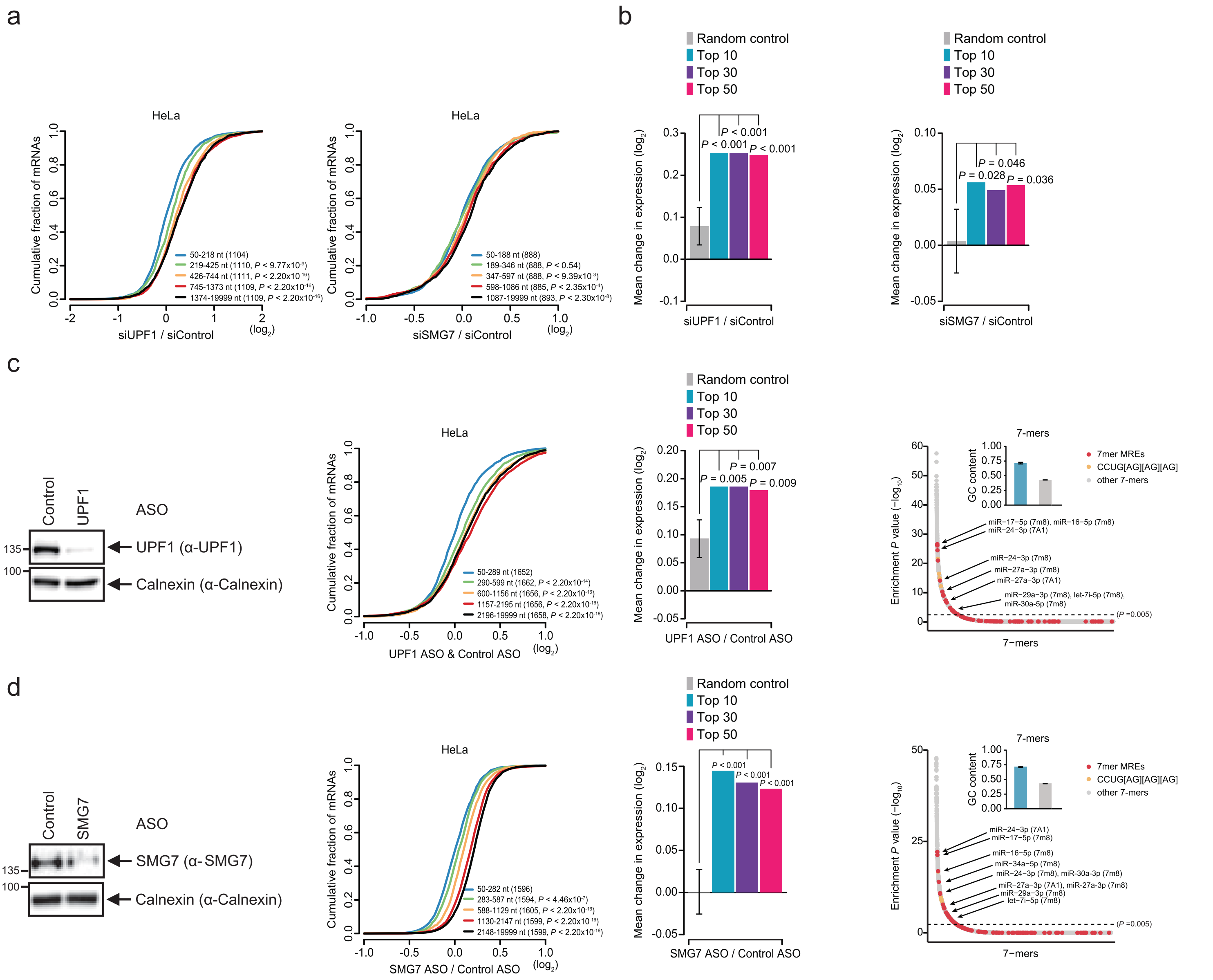

Supplementary Figure 9. Examination of siRNA off-target effects on UMD targeting. (a) Cumulative fractions of log2 changes in the expression of genes in siUPF1-treated (left) and siSMG7-treated (right) cells against siControl-treated cells are shown as CDF graphs of our HeLa cell data after excluding 6-mer off-targets of siRNAs. Otherwise, as in Fig. 1b. (b) Mean changes in the expression (log2 scale) of dEJ-free mRNAs (excluding 6-mer off-targets) embedding 7-mer target sites of random controls (gray) and the 10 (blue), 30 (purple), and 50 (red) most abundant miRNA families under siUPF1-treated (left) and siSMG7-treated (right) conditions. Otherwise, as in Fig. 3a. (c, d) ASO-mediated UPF1 and SMG7 downregulation and bioinformatics analyses. HeLa cells were transiently transfected with ASOs for the downregulation of UPF1 with a control (c) and for the downregulation of SMG7 with a control (d). Western blotting (WB; left-most) and RNA-seq were performed to test the downregulation of proteins and to quantify the transcripts, respectively. Cumulative fractions of log2 changes in the expression of genes in UPF1 ASO-treated versus control ASO-treated cells (c) and in SMG7 ASO-treated and control ASO-treated cells (d) are shown as CDF graphs. Mean changes in the expression (log2 scale) of dEJ-free mRNAs embedding 7-mer target sites of random controls (gray) and the 10 (blue), 30 (purple), and 50 (red) most abundant miRNA families under UPF1 ASO-treated (c) and siSMG7-treated (d) conditions. Analyses of all possible 7-mers enriched in the 3′UTR of UMD targets under UPF1 ASO-treated (c) and SMG7 ASO-treated (d) conditions.

Supplementary Table 1. UPF1-dependent mRNA decay (UMD) mRNAs information

RNA-seq	Cell line	PTC-free mRNAs	UMD mRNAs	Proportion of UMD mRNAs (%)
Hurt et. al., 2013	mES	8,130	3,416	42.0%
Wang et. al., 2014	HeLa	7,732	4,127	53.4%
Tani et. al., 2012	HeLa	4,917	2,478	50.4%
ENCODE, 2016	K562	6,863	2,548	37.1%
Our experiment	HeLa	7,117	3,419	48.0%

Supplementary Table 2. Top 50 most abundant endo-miRNA seed families; Related to Figures 2,3,5,6,7

HeLa				mES			
Rank	Representative miRNA	Seed sequence	miRNA seed family	Rank	miRNA seed family (mES)	Seed sequence	miRNA seed family
1	hsa-miR-21-5p	AGCUUA	hsa-miR-21,hsa-miR-590-5p	1	mmu-miR-466m-3p	ACAUAC	mmu-miR-1b-5p,mmu-miR-466m-3p,mmu-miR-466o-3p
2	hsa-let-7i-5p	GAGGUA	hsa-let-7i,hsa-let-7a,hsa-let-7f,hsa-let-7c,hsa-let-7b,hsa-let-7g,hsa-let-7e,hsa-let-7d,hsa-miR-98	2	mmu-miR-290	CUCAAA	mmu-miR-290a-5p,mmu-miR-292a-5p,mmu-miR-293-5p,mmu-miR-294-5p,mmu-miR-295-5p,mmu-miR-292b-5p
3	hsa-miR-24-3p	GGCUCA	hsa-miR-24	3	mmu-miR-291a-5p	AUCAAA	mmu-miR-291a-5p,mmu-miR-291b-5p
4	hsa-miR-27a-3p	UCACAG	hsa-miR-27a,hsa-miR-27b	4	mmu-miR-294	AAGUGC	mmu-miR-290a-3p,mmu-miR-291a-3p,mmu-miR-292a-3p,mmu-miR-294-3p,mmu-miR-295-3p,mmu-miR-302a-3p,mmu-miR-350-5p,mmu-miR-467a-5p,mmu-miR-291b-3p,mmu-miR-302b-3p,mmu-miR-302d-3p,mmu-miR-467c-5p,mmu-miR-467d-5p
5	hsa-miR-17-5p	AAAGUG	hsa-miR-17,hsa-miR-20a,hsa-miR-106b,hsa-miR-93,hsa-miR-106a,hsa-miR-20b	5	mmu-miR-293	GUGCCG	mmu-miR-293-3p
6	hsa-miR-16-5p	AGCAGC	hsa-miR-16,hsa-miR-15a,hsa-miR-15b,hsa-miR-424,hsa-miR-195,hsa-miR-503,hsa-miR-497	6	mmu-miR-297a-3-3p	UAUACA	mmu-let-7a-1-3p,mmu-let-7b-3p,mmu-let-7c-2-3p,mmu-let-7f-1-3p,mmu-let-7f-2-3p,mmu-miR-98-3p,mmu-miR-467b-3p,mmu-miR-467c-3p,mmu-miR-467d-3p,mmu-miR-467e-3p,mmu-miR-467f,mmu-miR-669l-3p,mmu-miR-669m-3p,mmu-miR-669d-2-3p
7	hsa-miR-26a-5p	UCAAGU	hsa-miR-26a,hsa-miR-26b	7	mmu-miR-466l-3p	AUAAAU	mmu-miR-466l-3p
8	hsa-miR-30a-5p	GUAAAC	hsa-miR-30a,hsa-miR-30c,hsa-miR-30d,hsa-miR-30e,hsa-miR-30b	8	mmu-miR-302c-5p	CUUUAA	mmu-miR-302b-5p,mmu-miR-302c-5p,mmu-miR-302d-5p,mmu-miR-3094-3p
9	hsa-miR-191-5p	AACGGA	hsa-miR-191	9	mmu-miR-466l-5p	UGUGUG	mmu-miR-466l-5p,mmu-miR-466n-5p
10	hsa-miR-22-3p	AGCUGC	hsa-miR-22	10	mmu-miR-669e	GUCUUG	mmu-miR-431-5p,mmu-miR-669e-5p
11	hsa-miR-92a-3p	AUUGCA	hsa-miR-92a,hsa-miR-25,hsa-miR-92b,hsa-miR-32	11	mmu-miR-20b	AAAGUG	mmu-miR-106a-5p,mmu-miR-106b-5p,mmu-miR-20a-5p,mmu-miR-93-5p,mmu-miR-17-5p,mmu-miR-20b-5p,mmu-miR-6383
12	hsa-miR-19b-3p	GUGCAA	hsa-miR-19b,hsa-miR-19a	12	mmu-miR-466m-5p	GUGUGC	mmu-miR-466h-5p,mmu-miR-466j,mmu-miR-669m-5p,mmu-miR-466m-5p
13	hsa-miR-130a-3p	AGUGCA	hsa-miR-130a,hsa-miR-301a,hsa-miR-454,hsa-miR-130b,hsa-miR-301b	13	mmu-miR-669o-5p	AGUUGU	mmu-miR-669o-5p
14	hsa-miR-29a-3p	AGCACC	hsa-miR-29a,hsa-miR-29b,hsa-miR-29c	14	mmu-miR-466c-5p	GAUGUG	mmu-miR-466b-5p,mmu-miR-466c-5p,mmu-miR-466o-5p
15	hsa-miR-125b-5p	CCCUGA	hsa-miR-125b,hsa-miR-125a-5p	15	mmu-miR-669b	GUUUUG	mmu-miR-19b-2-5p,mmu-miR-19b-1-5p,mmu-miR-669b-5p
16	hsa-miR-103a-3p	GCAGCA	hsa-miR-103,hsa-miR-107	16	mmu-miR-669a-5p	GUUGUG	mmu-miR-669a-5p,mmu-miR-669f-5p,mmu-miR-669l-5p,mmu-miR-669p-5p
17	hsa-miR-23a-3p	UCACAU	hsa-miR-23a,hsa-miR-23b	17	mmu-miR-466f	CGUGUG	mmu-miR-696,mmu-miR-466f
18	hsa-miR-186-5p	AAAGAA	hsa-miR-186	18	mmu-miR-669h	UGCAUG	mmu-miR-669h-5p
19	hsa-miR-100-5p	ACCCGU	hsa-miR-100,hsa-miR-99a,hsa-miR-99b	19	mmu-miR-363-5p	AGGUGG	mmu-miR-363-5p,mmu-miR-7037-5p
20	hsa-miR-33a-5p	UGCAUU	hsa-miR-33a,hsa-miR-33b	20	mmu-miR-669d	CUUGUG	mmu-miR-669d-5p
21	hsa-miR-320a	AAAGCU	hsa-miR-320a,hsa-miR-320b,hsa-miR-320c,hsa-miR-320d	21	mmu-miR-466a-5p	AUGUGU	mmu-miR-466a-5p,mmu-miR-466e-5p,mmu-miR-511-3p,mmu-miR-1187,mmu-miR-1198-5p,mmu-miR-466p-5p
22	hsa-miR-181a-5p	ACAUUC	hsa-miR-181a,hsa-miR-181b,hsa-miR-181c,hsa-miR-181d	22	mmu-miR-92a-2	AUUGCA	mmu-miR-92a-3p,mmu-miR-25-3p,mmu-miR-32-5p,mmu-miR-363-3p,mmu-miR-367-3p,mmu-miR-92b-3p
23	hsa-miR-193b-3p	ACUGGC	hsa-miR-193b,hsa-miR-193a-3p	23	mmu-miR-18b	AAGGUG	mmu-miR-18a-5p,mmu-miR-18b-5p
24	hsa-miR-143-3p	GAGAUG	hsa-miR-143	24	mmu-miR-669c	UAGUUG	mmu-miR-669c-5p
25	hsa-miR-18a-5p	AAGGUG	hsa-miR-18a,hsa-miR-18b	25	mmu-miR-466d-5p	GUGUGU	mmu-miR-466d-5p,mmu-miR-466i-5p,mmu-miR-466k,mmu-miR-6903-3p
26	hsa-miR-196a-5p	AGGUAG	hsa-miR-196a,hsa-miR-196b	26	mmu-miR-297a-5-3p	UGUAUG	mmu-miR-297a-5p,mmu-miR-675-3p,mmu-miR-297b-5p,mmu-miR-297c-5p
27	hsa-miR-101-3p	ACAGUA	hsa-miR-101	27	mmu-miR-543	AACAUU	mmu-miR-543-3p,mmu-miR-7210-5p
28	hsa-miR-423-3p	GCUCGG	hsa-miR-423-3p	28	mmu-miR-466a-3p	AUACAU	mmu-miR-466a-3p,mmu-miR-297b-3p,mmu-miR-297a-3p,mmu-miR-297c-3p,mmu-miR-466e-3p,mmu-miR-466d-3p,mmu-miR-669d-3p,mmu-miR-467g,mmu-miR-466n-3p
29	hsa-miR-224-5p	AAGUCA	hsa-miR-224	29	mmu-miR-22	AGCUGC	mmu-miR-22-3p
30	hsa-miR-222-3p	GCUACA	hsa-miR-222,hsa-miR-221	30	mmu-miR-669a-2-3p	CAUAAC	mmu-miR-669a-3p,mmu-miR-669a-3-3p,mmu-miR-669o-3p
31	hsa-miR-148b-3p	CAGUGC	hsa-miR-148b,hsa-miR-148a,hsa-miR-152	31	mmu-miR-669k	AUGCAU	mmu-miR-669k-3p,mmu-miR-669h-3p
32	hsa-miR-365a-3p	AAUGCC	hsa-miR-365	32	mmu-miR-3091-5p	AUGGGU	mmu-miR-3091-5p
33	hsa-miR-378a-3p	CUGGAC	hsa-miR-378	33	mmu-miR-130b-3p	AGUGCA	mmu-miR-130a-3p,mmu-miR-301a-3p,mmu-miR-130b-3p,mmu-miR-301b-3p,mmu-miR-721,mmu-miR-6341,mmu-miR-130c,mmu-miR-6389
34	hsa-miR-151a-5p	CGAGGA	hsa-miR-151-5p	34	mmu-miR-3063	GAGGAA	mmu-miR-3063-3p,mmu-miR-6925-5p
35	hsa-miR-34a-5p	GGCAGU	hsa-miR-34a,hsa-miR-449a	35	mmu-miR-674-5p	CACUGA	mmu-miR-674-5p,mmu-miR-1947-3p,mmu-miR-3473d
36	hsa-miR-374a-5p	UAUAAU	hsa-miR-374a,hsa-miR-374b	36	mmu-miR-467e	UAAGUG	mmu-miR-668-5p,mmu-miR-467b-5p,mmu-miR-467e-5p,mmu-miR-467h,mmu-miR-5617-5p
37	hsa-miR-28-5p	AGGAGC	hsa-miR-28-5p,hsa-miR-708	37	mmu-miR-3086-3p	CCAAUG	mmu-miR-3086-3p
38	hsa-miR-96-5p	UUGGCA	hsa-miR-96,hsa-miR-182,hsa-miR-1271	38	mmu-miR-376c	UGGAUA	mmu-miR-376b-5p,mmu-miR-376c-5p
39	hsa-miR-151a-3p	UAGACU	hsa-miR-151-3p	39	mmu-miR-96	UUGGCA	mmu-miR-182-5p,mmu-miR-96-5p
40	hsa-miR-21-3p	AACACC	hsa-miR-21*	40	mmu-miR-495	AACAAA	mmu-miR-7a-1-3p,mmu-miR-495-3p,mmu-miR-1192
41	hsa-miR-145-5p	UCCAGU	hsa-miR-145	41	mmu-miR-337-3p	CAGCUC	mmu-miR-337-3p
42	hsa-miR-423-5p	GAGGGG	hsa-miR-423-5p	42	mmu-miR-1968	GCAGCU	mmu-miR-1968-5p
43	hsa-miR-484	CAGGCU	hsa-miR-484	43	mmu-miR-672	GAGGUU	mmu-miR-329-5p,mmu-miR-377-5p,mmu-miR-672-5p
44	hsa-miR-452-5p	ACUGUU	hsa-miR-452	44	mmu-miR-539-5p	GAGAAA	mmu-miR-539-5p,mmu-miR-706
45	hsa-miR-30a-3p	UUUCAG	hsa-miR-30a*,hsa-miR-30e*,hsa-miR-30d*	45	mmu-miR-1193-3p	AGGUCA	mmu-miR-370-5p,mmu-miR-1193-3p
46	hsa-miR-324-5p	GCAUCC	hsa-miR-324-5p	46	mmu-miR-758	UUGUGA	mmu-miR-153-5p,mmu-miR-758-3p
47	hsa-miR-582-5p	UACAGU	hsa-miR-582-5p	47	mmu-miR-299-5p	GGUUUA	mmu-miR-299a-5p
48	hsa-let-7i-3p	UGCGCA	hsa-let-7i*	48	mmu-miR-329-3p	ACACAC	mmu-miR-329-3p,mmu-miR-362-3p,mmu-miR-669c-3p
49	hsa-miR-361-5p	UAUCAG	hsa-miR-361-5p	49	mmu-miR-421	UCAACA	mmu-miR-421-3p
50	hsa-miR-155-5p	UAAUGC	hsa-miR-155	50	mmu-miR-3108	UCUCUA	mmu-miR-3108-5p

Supplementary Table 3. The signal to noise ratio of 3-mers in UMD 3’UTRs and sequences reverse complementary to human and conserved mammalian miRNAs; Related to Figure2

	3mer of UMD	Sequences reverse complementary to miRNAs from Fromm et al.				Sequences reverse complementary to conserved mammalian miRNAs			
Motif	Signal to noise ratio	Fromm et al., (1046)	Random (mean)	*P*	Signal to noise ratio	Fromm et al., & conserved miRNA (384)	Random (mean)	*P*	Signal to noise ratio
CAG	1.46	123	64.32	0.04	1.91	47	29.83	0.14	1.58
CTG	1.44	144	68.83	0.03	2.09	63	31.30	0.06	2.01
AAA	1.39	68	55.04	0.40	1.24	13	26.11	0.58	0.50
TTT	1.32	94	42.62	0.18	2.21	36	20.44	0.27	1.76
CCT	1.26	107	62.23	0.17	1.72	36	28.54	0.39	1.26
CCA	1.25	110	65.76	0.18	1.67	36	30.80	0.37	1.17
GGG	1.23	59	25.73	0.20	2.29	28	12.23	0.18	2.29
CCC	1.22	88	37.43	0.14	2.35	10	16.83	0.49	0.59
AGG	1.21	90	45.97	0.18	1.96	35	21.08	0.26	1.66
GAG	1.20	90	44.24	0.13	2.03	28	21.23	0.30	1.32
TGG	1.20	83	48.90	0.17	1.70	16	21.92	0.56	0.73
GGA	1.19	76	46.24	0.27	1.64	19	21.12	0.41	0.90
TGT	1.19	114	55.33	0.12	2.06	62	25.13	0.04	2.47
AGA	1.18	94	55.22	0.22	1.70	15	25.48	0.67	0.59
CTC	1.18	110	57.13	0.17	1.93	33	27.67	0.35	1.19
GCC	1.17	97	56.60	0.19	1.71	36	26.38	0.31	1.36
GTG	1.16	87	42.60	0.13	2.04	43	19.27	0.12	2.23
CAC	1.16	135	59.79	0.03	2.26	78	27.52	0.01	2.83
GAA	1.12	80	52.68	0.27	1.52	13	23.64	0.52	0.55
TCT	1.12	94	53.94	0.25	1.74	31	25.76	0.36	1.20
TCC	1.11	87	48.95	0.19	1.78	21	22.27	0.48	0.94
AGC	1.10	99	70.58	0.22	1.40	53	33.54	0.17	1.58
ACA	1.09	86	65.29	0.27	1.32	42	30.41	0.24	1.38
GGC	1.09	69	37.22	0.19	1.85	17	17.18	0.38	0.99
GCA	1.06	154	59.48	0.04	2.59	86	28.18	0.01	3.05
TGC	1.06	118	69.29	0.15	1.70	65	32.10	0.12	2.02
GCT	1.03	87	64.46	0.33	1.35	28	30.77	0.51	0.91
TTC	1.03	51	53.61	0.46	0.95	17	24.85	0.55	0.68
AAG	1.02	84	64.93	0.31	1.29	26	30.56	0.51	0.85
CTT	1.02	118	62.97	0.16	1.87	59	29.56	0.15	2.00
TCA	0.99	67	65.50	0.45	1.02	16	29.85	0.71	0.54
TGA	0.99	111	66.31	0.20	1.67	43	29.63	0.24	1.45
CAT	0.96	66	67.24	0.46	0.98	27	31.10	0.47	0.87
ATG	0.96	84	55.15	0.26	1.52	38	25.19	0.27	1.51
ACT	0.94	139	70.35	0.07	1.98	90	31.59	0.00	2.85
ACC	0.94	104	60.26	0.16	1.73	38	27.61	0.25	1.38
TTG	0.92	90	58.33	0.20	1.54	28	27.36	0.44	1.02
AAT	0.92	76	56.64	0.30	1.34	28	26.62	0.41	1.05
ATA	0.92	69	42.06	0.27	1.64	13	19.61	0.52	0.66
ATT	0.91	67	53.63	0.36	1.25	21	24.53	0.49	0.86
TAT	0.90	67	41.56	0.22	1.61	24	19.16	0.30	1.25
AGT	0.90	74	65.04	0.43	1.14	23	29.54	0.63	0.78
TAA	0.88	49	58.68	0.49	0.84	5	27.44	0.78	0.18
GAC	0.87	53	58.04	0.48	0.91	20	26.12	0.53	0.77
AAC	0.85	77	68.25	0.36	1.13	12	32.65	0.71	0.37
GTT	0.85	66	48.05	0.30	1.37	34	21.73	0.25	1.56
TTA	0.85	51	53.89	0.46	0.95	25	24.72	0.44	1.01
GGT	0.85	56	33.82	0.26	1.66	23	15.10	0.26	1.52
CAA	0.84	89	77.03	0.33	1.16	38	36.07	0.38	1.05
GTC	0.83	65	46.31	0.31	1.40	25	21.37	0.41	1.17
GTA	0.83	73	43.28	0.27	1.69	27	19.97	0.31	1.35
GAT	0.78	55	41.97	0.34	1.31	15	18.47	0.51	0.81
TAC	0.78	86	55.71	0.25	1.54	56	25.19	0.08	2.22
ATC	0.77	53	41.79	0.37	1.27	11	18.72	0.57	0.59
TAG	0.71	62	54.34	0.41	1.14	15	25.20	0.64	0.60
CTA	0.69	82	70.39	0.40	1.16	37	33.50	0.41	1.10
CCG	0.49	48	31.99	0.31	1.50	7	14.40	0.55	0.49
CGT	0.48	28	33.43	0.46	0.84	5	15.27	0.55	0.33
CGC	0.47	36	32.94	0.41	1.09	6	15.83	0.63	0.38
CGG	0.47	27	26.97	0.38	1.00	5	11.78	0.44	0.42
GCG	0.46	30	31.79	0.46	0.94	2	15.02	0.58	0.13
ACG	0.45	20	40.61	0.62	0.49	6	18.25	0.64	0.33
TCG	0.34	17	29.01	0.58	0.59	7	13.36	0.56	0.52
CGA	0.33	25	31.65	0.51	0.79	5	13.96	0.55	0.36

Supplementary Table 4. The signal to noise ratio of 3-mers in sequences reverse complementary to fly miRNAs; Related to Figure 2

Motif	Sequences reverese complementary to fly miRNAs (miRbase v.21)			
	miRBase & conserved miRNA (137)	Random (mean)	*P*	Signal to noise ratio
GGG	6	3.01	0.13	1.99
CTG	15	9.03	0.19	1.66
CCT	15	9.10	0.24	1.65
CGA	12	7.71	0.27	1.56
GAG	8	8.18	0.41	0.98
CGG	4	4.33	0.36	0.92
TCG	6	6.85	0.41	0.88
CCG	5	5.87	0.45	0.85
ACG	6	7.94	0.52	0.76
CGC	5	6.66	0.43	0.75
AGG	5	7.06	0.46	0.71
GGA	4	5.84	0.41	0.68
CGT	4	7.41	0.54	0.54
GCG	0	5.76	0.63	0.00

Supplementary Table 5. The signal to noise ratio of 3-mers in sequences reverse complementary to worm miRNAs; Related to Figure 2

Motif	Sequences reverse-complementary to conserved worm miRNAs (miRbase v.21)			
	miRBase & conserved miRNA (123)	Random (mean)	*P*	Signal to noise ratio
CGG	16	4.71	0.06	3.39
GGG	11	3.70	0.10	2.97
CCG	8	4.71	0.22	1.70
ACG	11	6.97	0.24	1.58
CTG	12	8.23	0.26	1.46
CCT	10	7.14	0.26	1.40
CGA	10	7.17	0.29	1.39
AGG	5	7.16	0.46	0.70
GAG	3	7.87	0.55	0.38
CGT	2	6.21	0.55	0.32
GCG	1	6.04	0.62	0.17
CGC	1	6.36	0.68	0.16
GGA	1	7.28	0.60	0.14
TCG	0	6.30	0.57	0.00

Supplementary Table 6. Knockdown of UPF1 and TNRC6A/C by RNAi; Related to Figure 7

Experiments	Expression values (FPKM)		
	TNRC6A	TNRC6C	UPF1
siTNRC6A/C & siUPF1	3.64	0.08	8.37
siTNRC6A/C	3.06	0.12	27.28
siUPF1	6.05	0.36	8.50
siControl	7.06	0.41	30.17

Supplementary Table 7. siRNAs and ASOs used in this study; Related to Figure 3,4,5,6,7

siRNA name	Sense (5' to 3')
Control	CCUACGCCACCAAUUUCGU
Dicer1-1	UGCUUGAAGCAGCUCUGGAUC
Dicer1-2	AAGGGCACCCAUCUCUAAUUA
SMG7-1	AACAGCACAGUCUACAAGCCA
SMG7-2	GCAAGAAACAUCUGUGAUA
TNRC6A	GCCUAAUCUCCGUGCUCAAGUGCCT
TNRC6C	GCAUUAAGUGCUAAACAAAUGGAT
UPF1	CCAAGAUGCAGUUCCGCUCCAUU

ASO name	Antisense (5' to 3')
UPF1-ASO-1	mA*mA*T*G*G*A*G*C*G*G*A*A*C*T*G*C*A*T*C*T*T*mG*mG
UPF1-ASO-2	mA*mG*A*A*T*A*A*G*A*T*G*C*T*G*A*T*G*G*A*C*T*A*A*mG*mC
SMG7-ASO-1	mC*mT*C*T*A*T*C*A*C*A*G*A*T*G*T*T*T*mC*mT
SMG7-ASO-2	mC*mT*G*G*C*T*T*G*T*A*G*A*C*T*G*T*G*mC*mT

*=Phosphorothioate bonds

mN=2'-O-Me base

Supplementary Table 8. qPCR primers used in this study; Related to Figure 4,5,6,7

qPCR primer name	Forward (5' to 3')	Reverse (5' to 3')
DNAL4	AGGGCTTTGGGTTTGAGATC	AGATGAGTGGCAGGAAAAGG
ATAD2B	AGAATTCCTCTTCAACCTGCC	CCTTGATATCGGCTCCACAG
RPS19BP1	GGAAGACGAAGGCAATTCAG	CGTCCTGGTCAGAAACTTCAG
PEA15	ACATCCCCAGCGAAAAGAG	ACCATAGTGAGTAGGTCAGGAC
CRAT	CAGCCTGTGGTCATCTACTC	AGGGTCTCGTTGTCAATCATG
ELL	AGCAAGGTGTCGGTGTTC	GATGTTGGAGAGGTAGAAGGAG
PAK4	AGCTGTCAGACTTTGGGTTC	GGGTGGCTCGTTGAAGTAG
DBNDD1	TGCCTATTCCTCCTGAGATCGT	GTGGACCTCCAGAGAGGAGAC
ZNF740	TGCGTTTCATCCAGAAGTACC	GCCTATAGAGAAAACTGCCCG
HPCAL1	GAACTTGGGCTCGGGAAG	AGAGGGAGTGGGTCAGAAC
MXI1	CTCAGGAGATGGAACGAATACG	ATGGGAGAACTCTGTGCTTTC
ATG4A	GCCCTTATCTGTAGACACTTGG	GCGTTGGTATTCTTTGGGTTG
RPA1	CTGTCCACACAGTGAAGAGGCC	CCCCTGGGTTCCCAATCACAT
ITM2B	ACATCAAGGCTGGAACCTATTTG	TGCGAAACAATTGCTGGCTT
GREB1L	GGCTGAGGTTCTGTTGCAGAGTG	TTACTCCTCACAAGAGTCACTGG
TXLNG	CCACTTCCCATAGAAGGCCACT	GTGACACGTGAATTGACCGTCC
REEP4	GCTGGGCCCAGGGTTCTATTTA	ATGTGCCAGGCCTTATGAAAGGC
LASP1	CTCCAGAGTCAGGTGCGCTA	CCATGCGGCTCTTCTCAAAC
EPAS1	CTGTATGGTCAGCTCAGCCC	GGCTGTCAGACCCGAAAAGA
HMGA1	CCAATAACAAGGAGCTCACCCTG	GCAGAACAGGAGGCAATGAGG
HUWE1	GGAAACCATCCCTGCCCTAC	TTTGTCCTGGGCTGCAATCT
TEX261	GGCCACCAGCAGGATCATAA	CCACCACTAGTCCACACGAC
ADD1	CCCCACAGTAGAGCACTTTTCAC	AACAACAGCATCTG CGAGGA
ATG16L1	GGGGTTGTGATGAAGGCCAAG	CAGCTCTAACACTCACATCCCC
FAM98A	TGAGTTAATACCAC GGCTCAGC	CAATAATAAAGGGCCTCCTGCAC
HTATIP2	GTCAACCTTAACACCCATCACCAA	CTCTGAACCACTGATGCAGAGCA
RANBP9	GAGCCCGAAGGACAAGTTCA	CACAGGCTGCTGGTATTGGA
LRRC40	TGAAAGATGGTGGGAGCAGAC	ACAGTCAGTGCAGGCAAGAG
Ezh2	GCTTCCTACATCGTAAGTGCAA	GCTCCCTCCAAATGCTGGTA
BDNF	ATCCACTGAGCAAAGCCGAA	CCTGGTGGAACATTGTGGCT
TNRC6A	ATGGCTGGTGTGGTGATGATA	TTCCCCTTTCCCTTCTCCTTTT
TNRC6C	TCCCCGCCATCATCTCAGAA	AACACTAGGTGCGGATGCAAT
UPF1	AATTTGGTTAAGAGACATGCGG	TCAGGGACCTTGATGACGTG
SMG7	GGCAGGCAGAAGTCCTGAAG	AGGCGTGATTCCAGAGATCC
FLuc	CCCCATCTTCGGCAACCAG	GCGCAAGAATAGCTCCTCC
RLuc	GTGGGCCACGACTGGGGGC	ACCATTTTCTCGCCCTCTTCGC
TAGLN	ACACAAGTCTTCACTCCTTCC	CACTGCACTATGATCCACTCC
S1PR2	AGTTCTGAAAGCCCCATGG	AACAGAGGATGACGATGAAGG
LOXL2	ACCGCATCTGGATGTACAAC	TGGCATTCGTTCAGACTCAG
STK36	GCTCTTCACCGTCTCTACTTTC	AAATTCCTCAGCTCCTTCTCTG

Supplementary Table 9. qPCR primers used in this study; Related to Supplementary Figure 3

qPCR primer name	Forward (5' to 3')
hsa-miR-21-5p	CGCTAGCTTATCAGACTGATGTTGA
hsa-let-7i-5p	CGCTGAGGTAGTAGTTTGTGCTGTT
hsa-miR-24-3p	TGGCTCAGTTCAGCAGGAACAG
hsa-miR-27a-3p	CGCTTCACAGTGGCTAAGTTCCGC
hsa-miR-17-5p	CGCCAAAGTGCTTACAGTGCAGGTAG
hsa-miR-16-5p	CGCTAGCAGCACGTAAATATTGGCG
hsa-miR-26a-5p	CGCTTCAAGTAATCCAGGATAGGC
hsa-miR-30a-3p	CGCCTTTCAGTCGGATGTTTGCAGC
hsa-miR-191-5p	CGCCAACGGAATCCCAAAAGCAGCTG

Supplementary Table 10. Cloning primers used in this study; Related to Figure 5 and Supplementary Figure 5

Cloning primer name	Sequences (5’ to 3’)
PEA15-5’	TACATGTTCCCAGCACAGTCTCTCCAGAGTACAGGTGGGTGGGGGCATCT
PEA15-middle-WT	GACTGTGCTGGGAACATGTACCACTGAGCCTGAGATGGGGATGAGGGCAG
PEA15-middle-Mut	GACTGTGCTGGGAACATGTACCATCAAGCCTGAGATGGGGATGAGGGCAG
PEA15-3’	AACTGAGTGG AAGAGGGGGC TCCCCTCTCT CTGCCCTCAT CCCCATCTCA
PEA15-XhoI-F	CGCCTCGAGAGATGCCCCCACCCACCTGT
PEA15-XbaI-R	GCGTCTAGAAACTGAGTGGAAGAGGGGGC
RPS19BP1-1-5'	TCCGCGGCTTCCTCGAGCCAGGCTGGTCCTGCATCGCCATCTGCTGGCCG
RPS19BP1-1-middle-WT	TGGCTCGAGGAAGCCGCGGAGCTGAGCCGAGTGGAGGCTGGAATGGAGCT
RPS19BP1-1-middle-Mut	TGGCTCGAGGAAGCCGCGGAGTCAAGCCGAGTGGAGGCTGGAATGGAGCT
RPS19BP1-1-3'	GTAAATCCTCCCCAGGACTTCCGGCCCACCAGCTCCATTCCAGCCTCCAC
RPS19BP1-1-XbaI-F	CGCTCTAGACGGCCAGCAGATGGCGATGC
RPS19BP1-1-XbaI-R	GCGTCTAGAGTAAATCCTCCCCAGGACTT
RPS19BP1-2-5'	GAATTAGCCTTGCTCCACAGCAAACAGCCCAGAGGCCCCACCTGGGCAAA
RPS19BP1-2-middle-WT	CTGTGGAGCAAGGCTAATTCCTGAGCCCTTGGGGACGACAGCTCCAGGAG
RPS19BP1-2-middle-Mut	CTGTGGAGCAAGGCTAATTCTCAAGCCCTTGGGGACGACAGCTCCAGGAG
RPS19BP1-2-3'	TTGTAACTTGGAAGCCCACCCTTCTTCCTACTCCTGGAGCTGTCGTCCCC
RPS19BP1-2-XbaI-F	CGCTCTAGATTTGCCCAGGTGGGGCCTCT
RPS19BP1-2-XbaI-R	GCGTCTAGATTGTAACTTGGAAGCCCACC
DNAL4-5'	GAAACTGGTACACAAAGACAAAAAGAAAAGACCCCAACTCTCCTTGGAAAAACCAGGACC
DNAL4-middle-WT	TGTCTTTGTGTACCAGTTTCCTGAGCCACGCCCAGTGTGTGAACTTGACA
DNAL4-middle-Mut	TGTCTTTGTGTACCAGTTTCCACTCCCACGCCCAGTGTGTGAACTTGACA
DNAL4-3'	GAGACTCCGAGGGAGACGGTTGAGAGCCTGGGGATGGAGATGTCAAGTTCACACACTGGG
DNAL4-XbaI-F	CGCCTCGAGGGTCCTGGTTTTTCCAAGGA
DNAL4-XbaI-R	GCGTCTAGAGAGACTCCGAGGGAGACGGT
ATAD2B-5'	GACTGCTCTGTGAGGAGCAGATTGGAGAGGATAAACCACTCATCTTGAAAAGT
ATAD2B-middle-WT	CAATCTGCTCCTCACAGAGCAGTCTTCTGAGCCATTCAATTTCAAATTGCACCAATTATG
ATAD2B-middle-Mut	CAATCTGCTCCTCACAGAGCAGTCTTCACTCCCATTCAATTTCAAATTGCACCAATTATG
ATAD2B-3'	AAAGAATGAGTGAGAGAGCACTTTACACCAAGGCTCTGCACATAATTGGTGCAATTTGAA
ATAD2B-XbaI-F	CGCCTCGAGACTTTTCAAGATGAGTGGT
ATAD2B-XbaI-R	GCGTCTAGAAAAGAATGAGTGAGAGAGCA
ELL-5'	CATCTTCCTCGGGTTTATTTTTTTAAATAAATCCTCTCTCACCGCCTTTTGCTCCCCCGA
ELL-middle-WT	AAATAAACCCGAGGAAGATGCTCATCTGAGCCAGCACCGCCGGCTTTCAGGGCAGCCCCT
ELL-middle-Mut	AAATAAACCCGAGGAAGATGCTCATCACTCCCAGCACCGCCGGCTTTCAGGGCAGCCCCT
ELL-3'	GAGGTGGGCTTGCAGCCACCCGCCAGGGCCAGACGTCTGCAGGGGCTGCCCTGAAAGCCG
ELL-XbaI-F	CGCCTCGAGTCGGGGGAGCAAAAGGCGGT
ELL-XbaI-R	GCGTCTAGAGAGGTGGGCTTGCAGCCACC
PAK4-5'	GGGGGCTGGGTGTCAGGCAAGGCGCCAGACACCCCTTGCAGGCTGGAGGACGGGGTGGCT
PAK4-middle-WT	TTGCCTGACACCCAGCCCCCTCTCCCCCTGAGCCATTGTGGGGGTCGATCATGAATGTCC
PAK4-middle-Mut	TTGCCTGACACCCAGCCCCCTCTCCCCCACTCCCATTGTGGGGGTCGATCATGAATGTCC
PAK4-3'	CAGAAAGGGGGCGCAGGGCTACGGGAAAAGGCCACTCTTCGGACATTCATGATCGACCCC
PAK4-XbaI-F	CGCCTCGAGAGCCACCCCGTCCTCCAGCC
PAK4-XbaI-R	GCGTCTAGACAGAAAGGGGGCGCAGGGCT
DBNDD1-5'	GCAGGTACACAGAAGGAGCTCAGGGCATGCCTGGGGCATCTCCAAAGCTCTGCTGAGAGT
DBNDD1-middle-WT	AGCTCCTTCTGTGTACCTGCTCCCACTTCTGAGCCACCCGCTGCCCCTCCGCACTGCTGG
DBNDD1-middle-Mut	AGCTCCTTCTGTGTACCTGCTCCCACTTCACTCCCACCCGCTGCCCCTCCGCACTGCTGG
DBNDD1-3'	ACCAGGGAAGGAGACCTGGCTGAGGCAGGAACTGGGTTTGCCAGCAGTGCGGAGGGGCAG
DBNDD1-XbaI-F	CGCCTCGAGACTCTCAGCAGAGCTTTGGA
DBNDD1-XbaI-R	GCGTCTAGAACCAGGGAAGGAGACCTGGC
ZNF740-5'	TCCTGCAAGCATACAAATACATGCATTCATATCTCACACACACTCTCTCTCACACACACA
ZNF740-middle-WT	GTATTTGTATGCTTGCAGGAGATGGCATATTATTGAGCCAAACCCTTCTCTGGCCCTCAC
ZNF740-middle-Mut	GTATTTGTATGCTTGCAGGAGATGGCATATTATTCTCGCAAACCCTTCTCTGGCCCTCAC
ZNF740-3'	TGGGCAAGAGGAGGCTATTTTCCACTCATCCTCCCTGAAGGTGAGGGCCAGAGAAGGGTT
ZNF740-XbaI-F	CGCCTCGAGTGTGTGTGTGAGAGAGAGTG
ZNF740-XbaI-R	GCGTCTAGATGGGCAAGAGGAGGCTATTT
SMG7 WT-1-ClaI-F	GCGATCGATGAGCCTGCAGAGCGCGCAG
SMG7 WT-3276-KpnI-R	GCGGGTACCTTATCAGTGTGGAGGGTTCATGGT
SMG7 Mut-1896-KpnI-R	GCGGGTACCTTATCAAGAAAATGCCACAGGCGG
