# Supplementary material for: UPF1/SMG7-dependent microRNA-mediated gene regulation
Source: Nat Commun. 2019 Sep 13;10:4181. doi: 10.1038/s41467-019-12123-7 (PMC6744440; doi:10.1038/s41467-019-12123-7)
Supplement: Supplementary file 3 — Description of Additional Supplementary Files [file 41467_2019_12123_MOESM3_ESM.pdf]

Description of Additional Supplementary Files

File Name: Supplementary Data 1
Description: Enriched 7mer MREs ($P < 0.005$); Related to Figure 2
