# Supplementary material for: UPF1/SMG7-dependent microRNA-mediated gene regulation
Source: Nat Commun. 2019 Sep 13;10:4181. doi: 10.1038/s41467-019-12123-7 (PMC6744440; doi:10.1038/s41467-019-12123-7)
Supplement: Supplementary file 5 — Reporting Summary [file 41467_2019_12123_MOESM5_ESM.pdf]

nature research

Corresponding author(s): Jungwook Hwang and Jin-Wu Nam

Last updated by author(s): Jul 22, 2018

Reporting Summary

Nature Research wishes to improve the reproducibility of the work that we publish. This form provides structure for consistency and transparency in reporting. For further information on Nature Research policies, see Authors & Referees and the Editorial Policy Checklist.

Statistics

For all statistical analyses, confirm that the following items are present in the figure legend, table legend, main text, or Methods section.

n/a	Confirmed	
☐	☒	The exact sample size (*n*) for each experimental group/condition, given as a discrete number and unit of measurement
☐	☒	A statement on whether measurements were taken from distinct samples or whether the same sample was measured repeatedly
☐	☒	The statistical test(s) used AND whether they are one- or two-sided<br>*Only common tests should be described solely by name; describe more complex techniques in the Methods section.*
☒	☐	A description of all covariates tested
☐	☒	A description of any assumptions or corrections, such as tests of normality and adjustment for multiple comparisons
☐	☒	A full description of the statistical parameters including central tendency (e.g. means) or other basic estimates (e.g. regression coefficient) AND variation (e.g. standard deviation) or associated estimates of uncertainty (e.g. confidence intervals)
☒	☐	For null hypothesis testing, the test statistic (e.g. *F*, *t*, *r*) with confidence intervals, effect sizes, degrees of freedom and *P* value noted<br>*Give P values as exact values whenever suitable.*
☒	☐	For Bayesian analysis, information on the choice of priors and Markov chain Monte Carlo settings
☒	☐	For hierarchical and complex designs, identification of the appropriate level for tests and full reporting of outcomes
☒	☐	Estimates of effect sizes (e.g. Cohen's *d*, Pearson's *r*), indicating how they were calculated

Our web collection on statistics for biologists contains articles on many of the points above.

Software and code

Policy information about availability of computer code

Data collection	No software was use for data collection.
Data analysis	- For transcriptome assembly, RNA-seq data from UPF1-depleted HeLa cells were mapped to the genomes (hg19: GRCh37.66 for humans; mm9 for mice) using TopHat (version 2.0.6). The mapped reads were subjected to Cufflinks (version 2.1.1) for reference-based transcriptome assembly (parameters: --GTF-guide, --min-intron-length 61, and --max-intron-length 265,006).<br>- Our RNA-seq data were trimmed by 9 and 6 nt for the first and second fragments of paired-end reads, respectively, to remove the adaptor sequence using seqtk version 1.0. All RNA-seq reads were trimmed with a minimum quality of 20, and reads less than 20 nt in length were removed using Sickle version 1.2 (parameters: -q 20 -l 20). Reads were mapped to the reference genomes (hg19 for human and mm9 for mouse) using TopHat version 2.0.6 80, allowing at most five genomic loci mapping, two nucleotide mismatches, and novel introns within 1–99th percentile length of known introns, but not allowing any mismatch at splice sites (parameters: solexa1.3-quals, splice-mismatches 0, min-intron-length 61, max-intron-length 265006, max-multi hits 5, read-mismatches 2). Expression levels and fragments per kilobase of transcript per million mapped reads (FPKM) were calculated by Cufflinks version 2.1.1 81. For mRNAs with multiple isoforms, we chose the isoform with the longest 3′UTR to measure its expression. Raw CLIP-seq data of HeLa cells were mapped to the human reference genome (hg19) using Bowtie version 4.4.7 82, allowing two mismatches but not allowing multi-loci mapping (parameters: v 2, m 1, best, strata). All replicates were combined using Bed tools version 2.17.0 83. To identify Ago2- or UPF1-binding sites in the 3′UTR of mRNAs, the findPeaks program of HOMER package 84 was used. For mES cells, Ago2- and UPF1-binding sites processed from CLIP-seq data were analyzed.<br>- The distribution of the values was transformed to a cumulative distribution function (CDF) to evaluate the statistical significance of the difference among groups from different 3'UTR bins using R function, ecdf (version 3.1.2). Statistical significance was tested by the K-S test.<br>- All custom python and R codes used in this manuscript are available on GitHub repository (https://github.com/jwnam/UPF1).

For manuscripts utilizing custom algorithms or software that are central to the research but not yet described in published literature, software must be made available to editors/reviewers. We strongly encourage code deposition in a community repository (e.g. GitHub). See the Nature Research guidelines for submitting code & software for further information.

Data

Policy information about availability of data

All manuscripts must include a data availability statement. This statement should provide the following information, where applicable:

- Accession codes, unique identifiers, or web links for publicly available datasets
- A list of figures that have associated raw data
- A description of any restrictions on data availability

Raw RNA-seq data have been deposited in the NCBI Gene Expression Omnibus (GEO; https://www.ncbi.nlm.nih.gov/geo/) under accession number GSE99169.

Field-specific reporting

Please select the one below that is the best fit for your research. If you are not sure, read the appropriate sections before making your selection.

☒ Life sciences ☐ Behavioural & social sciences ☐ Ecological, evolutionary & environmental sciences

For a reference copy of the document with all sections, see nature.com/documents/nr-reporting-summary-flat.pdf

Life sciences study design

All studies must disclose on these points even when the disclosure is negative.

Sample size	At least three samples were analyzed. Although there is a clear difference, if a statistical significance is marginal, additional samples were performed.
Data exclusions	No data exclusion.
Replication	All attempt replications were successful.
Randomization	Not applicable.
Blinding	Not applicable; All experiments were based on cell lines; For RNAi, we have to open which one is control to remove off-targets properly.

Reporting for specific materials, systems and methods

We require information from authors about some types of materials, experimental systems and methods used in many studies. Here, indicate whether each material, system or method listed is relevant to your study. If you are not sure if a list item applies to your research, read the appropriate section before selecting a response.

Materials & experimental systems

n/a	Involved in the study	
☐	☒	Antibodies
☐	☒	Eukaryotic cell lines
☒	☐	Palaeontology
☒	☐	Animals and other organisms
☒	☐	Human research participants
☒	☐	Clinical data

Methods

n/a	Involved in the study	
☒	☐	ChIP-seq
☒	☐	Flow cytometry
☒	☐	MRI-based neuroimaging

Antibodies

Antibodies used	FLAG (GenScript, Piscataway, NJ, USA, A00187-100), UPF1 (Cell Signaling Technology, Danvers, MA, USA, #9435), Dicer (Cell Signaling Technology, #5362), SMG7 (Bethyl Laboratories, Montgomery, TX, USA, A302-170A), β-actin (Sigma, A2228), PABP (Santa Cruz Biotechnology Dallas, TX, USA, sc-28834), Ago2 (Abnova, Taipei, Taiwan, H00027161-M01), NOT1 (Proteintech, Rosemont, IL, USA, 14276-1-AP), NOT3 (Abcam, Cambridge, Cambridgeshire, UK, ab154276), TNRC6A (Abcam, Cambridge, Cambridgeshire, UK, ab156173) TNRC6C (Bethyl Laboratories, Montgomery, TX, USA, A303-969A) and Calnexin (Santa Cruz Biotechnology, Dallas, TX, USA, sc-11397).
Validation	All antibodies were purchased from the commercial companies with catalog numbers listed above.

Eukaryotic cell lines

Policy information about cell lines

Cell line source(s)	HeLa cells (KCLB #1002)
Authentication	Cell line was purchased from Korean Cell Line Bank.
Mycoplasma contamination	Cell line was tested negative for mycoplasma contamination by Lilif Diagnostics (e-Myco Mycoplasma PCR Detection Kit, Cat#25235).
Commonly misidentified lines (See ICLAC register)	*Name any commonly misidentified cell lines used in the study and provide a rationale for their use.*
